# Virus–virus interactions impact the population dynamics of influenza and the common cold

**DOI:** 10.1073/pnas.1911083116

**Published:** 2019-12-16

**Authors:** Sema Nickbakhsh, Colette Mair, Louise Matthews, Richard Reeve, Paul C. D. Johnson, Fiona Thorburn, Beatrix von Wissmann, Arlene Reynolds, James McMenamin, Rory N. Gunson, Pablo R. Murcia

**Affiliations:** ^a^MRC-University of Glasgow Centre for Virus Research, Institute of Infection, Immunity and Inflammation, College of Medical, Veterinary and Life Sciences, University of Glasgow, G61 1QH Glasgow, United Kingdom;; ^b^School of Mathematics and Statistics, College of Science and Engineering, University of Glasgow, G12 8QQ Glasgow, United Kingdom;; ^c^Boyd Orr Centre for Population and Ecosystem Health, Institute of Biodiversity, Animal Health and Comparative Medicine, College of Medical, Veterinary and Life Sciences, University of Glasgow, G12 8QQ Glasgow, United Kingdom;; ^d^The Queen Elizabeth University Hospital, NHS Greater Glasgow and Clyde, G51 4TF Glasgow, United Kingdom;; ^e^Public Health, NHS Greater Glasgow and Clyde, G12 0XH Glasgow, United Kingdom;; ^f^Health Protection Scotland, NHS National Services Scotland, G2 6QE Glasgow, United Kingdom;; ^g^West of Scotland Specialist Virology Centre, NHS Greater Glasgow and Clyde, G31 2ER Glasgow, United Kingdom

**Keywords:** epidemiology, virology, ecology

## Abstract

The human respiratory tract hosts a diverse community of cocirculating viruses that are responsible for acute respiratory infections. This shared niche provides the opportunity for virus–virus interactions which have the potential to affect individual infection risks and in turn influence dynamics of infection at population scales. However, quantitative evidence for interactions has lacked suitable data and appropriate analytical tools. Here, we expose and quantify interactions among respiratory viruses using bespoke analyses of infection time series at the population scale and coinfections at the individual host scale. We analyzed diagnostic data from 44,230 cases of respiratory illness that were tested for 11 taxonomically broad groups of respiratory viruses over 9 y. Key to our analyses was accounting for alternative drivers of correlated infection frequency, such as age and seasonal dependencies in infection risk, allowing us to obtain strong support for the existence of negative interactions between influenza and noninfluenza viruses and positive interactions among noninfluenza viruses. In mathematical simulations that mimic 2-pathogen dynamics, we show that transient immune-mediated interference can cause a relatively ubiquitous common cold-like virus to diminish during peak activity of a seasonal virus, supporting the potential role of innate immunity in driving the asynchronous circulation of influenza A and rhinovirus. These findings have important implications for understanding the linked epidemiological dynamics of viral respiratory infections, an important step towards improved accuracy of disease forecasting models and evaluation of disease control interventions.

## Significance

When multiple pathogens cocirculate this can lead to competitive or cooperative forms of pathogen–pathogen interactions. It is believed that such interactions occur among cold and flu viruses, perhaps through broad-acting immunity, resulting in interlinked epidemiological patterns of infection. However, to date, quantitative evidence has been limited. We analyzed a large collection of diagnostic reports collected over multiple years for 11 respiratory viruses. Our analyses provide strong statistical support for the existence of interactions among respiratory viruses. Using computer simulations, we found that very short-lived interferences may explain why common cold infections are less frequent during flu seasons. Improved understanding of how the epidemiology of viral infections is interlinked can help improve disease forecasting and evaluation of disease control interventions.

The human respiratory tract hosts a community of viruses that cocirculate in time and space, and as such it forms an ecological niche. Shared niches are expected to facilitate interspecific interactions which may lead to linked population dynamics among distinct pathogen species (1, 2). In the context of respiratory infections, a well-known example is the coseasonality of influenza and pneumococcus, driven by an enhanced susceptibility to secondary bacterial colonization subsequent to influenza infection (3, 4). Indeed, respiratory bacteria–bacteria and bacteria–virus interactions, and the underlying mechanisms by which they arise, are extensively studied (5–8). In contrast, evidence for the occurrence of virus–virus interactions remains scarce and the potential mechanisms are elusive, demanding greater research attention. The occurrence of such interactions may have profound economic implications, if the circulation of one pathogen enhances or diminishes the infection incidence of another, through impacts on the healthcare burden, public health planning, and the clinical management of respiratory illness.

Early interest in the potential for interference among taxonomically distinct groups of respiratory viruses stemmed from epidemiological observations of the temporal patterns of infection in respiratory virus outbreaks (9–11). More recently, the influenza A virus (IAV) pandemic of 2009 further galvanized interest in the epidemiological interactions among respiratory viruses. It was postulated that rhinovirus (RV) may have delayed the introduction of the pandemic virus into Europe (12, 13), while the pandemic virus may have, in turn, interfered with epidemics of respiratory syncytial virus (RSV) (14, 15). Clinical studies utilizing coinfection data from diagnostic tests have also suggested virus–virus interference at the host scale (13, 16, 17).

The role of adaptive immunity in driving virus interferences that alter the population dynamics of antigenically similar virus strains is well known (18, 19). For example, antibody-driven cross-immunity is believed to restrict influenza virus strain diversity, leading to sequential strain replacement over time (20). Such antibody-driven virus interactions might even shape the temporal patterns of RSV, human parainfluenza virus (PIV), and human metapneumovirus (MPV) infections, which are taxonomically grouped into the same virus family (21). However, where antigenically distant virus groups are concerned, the mechanisms are obscure, although the variety of possibilities includes innate immunity, resource competition, and other cellular processes (22–25). Recent experimental models of respiratory virus coinfections have demonstrated several interaction-induced effects, from enhanced (26) or reduced (22, 23) viral growth to the attenuation of disease (23, 24). It has also been shown that cell fusion induced by certain viruses may enhance the replication of others in coinfections (26).

However, despite epidemiological, clinical, and experimental indications of interactions among respiratory viruses, quantitatively robust evidence is lacking. Such evidence has been hard to acquire, due to a paucity of consistently derived epidemiological time-series data over a sufficient time frame to provide well-powered analyses, and the lack of analytical approaches to differentiate true virus–virus interactions from other drivers of coseasonality, such as age-dependent host mixing patterns (27, 28) and environmental factors associated with virus survival and transmission (29–31). As a result, studies evaluating virus–virus interactions from epidemiological time series have thus far not accounted for such alternative drivers of coseasonality.

Here, we apply a series of statistical approaches and provide robust statistical evidence for the existence of interactions among respiratory viruses. We examined virological diagnostic data from 44,230 episodes of respiratory illness accrued over a 9-y time frame in a study made possible by the implementation of multiplex-PCR methods in routine diagnostics that allow the simultaneous detection of multiple viruses from a single respiratory specimen. Each patient was tested for 11 virus groups (28, 29), providing a single, coherent data source for the epidemiological examination of infection dynamics of both cocirculating viruses in general and coinfection patterns in individual patients. Using these data, our study addresses the following questions: 1) Is there statistical evidence of virus–virus interactions in the temporal patterns of infection at the population scale?, 2) Is there statistical evidence of virus–virus interactions in coinfection patterns at the individual host scale?, and 3) Is a short-lived immune-mediated interference at the scale of individual hosts sufficient to induce asynchronous seasonal patterns of infection at the population scale?

## Results

### The Overall Prevalence of Any Viral Respiratory Infection among Patients with Respiratory Illness Is Relatively Stable over Time, Despite Strongly Varying Prevalences of Individual Viruses.

**Fig. 1.**
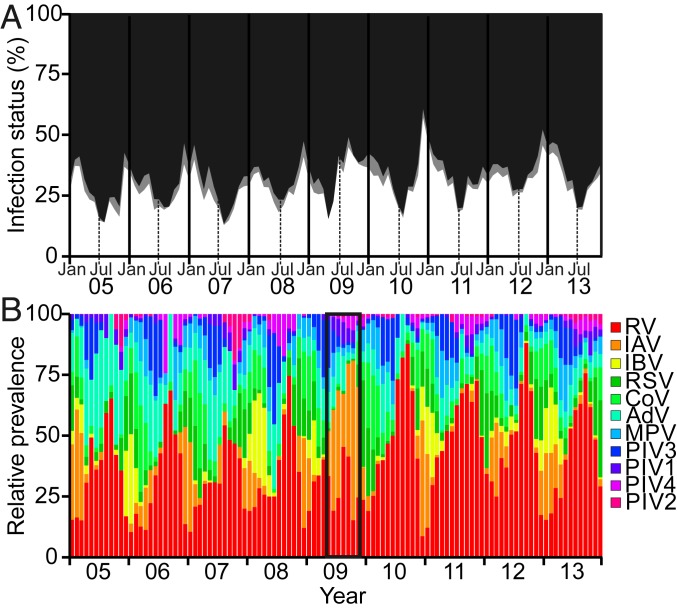
Temporal patterns of viral respiratory infections detected among patients in Glasgow, United Kingdom, 2005 to 2013. (*A*) Percentage of patients diagnosed with a single viral infection (white), a viral coinfection (gray), or determined to be virus-negative (black) by multiplex RT-PCR in each calendar month from 2005 to 2013 (6-mo intervals depicted by vertical lines; Jan = January, Jul = July). (*B*) Relative virus prevalences in each calendar month, from 2005 to 2013; note total virus counts may sum to more than those informing single infection prevalences due to coinfections, and test frequency denominators vary slightly across viruses. During the first wave of the United Kingdom’s influenza A pandemic [A(H1N1)pdm09] in 2009, infections with influenza A virus were relatively more prevalent among the patient population than noninfluenza virus infections (highlighted by black box). RV = rhinoviruses (A–C); IAV = influenza A virus (H1N1 and H3N2); IBV = influenza B virus; RSV = respiratory syncytial virus; CoV = human coronaviruses (229E, NL63, HKU1); AdV = human adenoviruses; MPV = human metapneumovirus; PIV3 = parainfluenza 3 virus; PIV1 = parainfluenza 1 virus; PIV4 = parainfluenza 4 virus; PIV2 = parainfluenza 2 virus. See also Table 1. Virus groups are listed in descending order of their total prevalence.

**Fig. 2.**
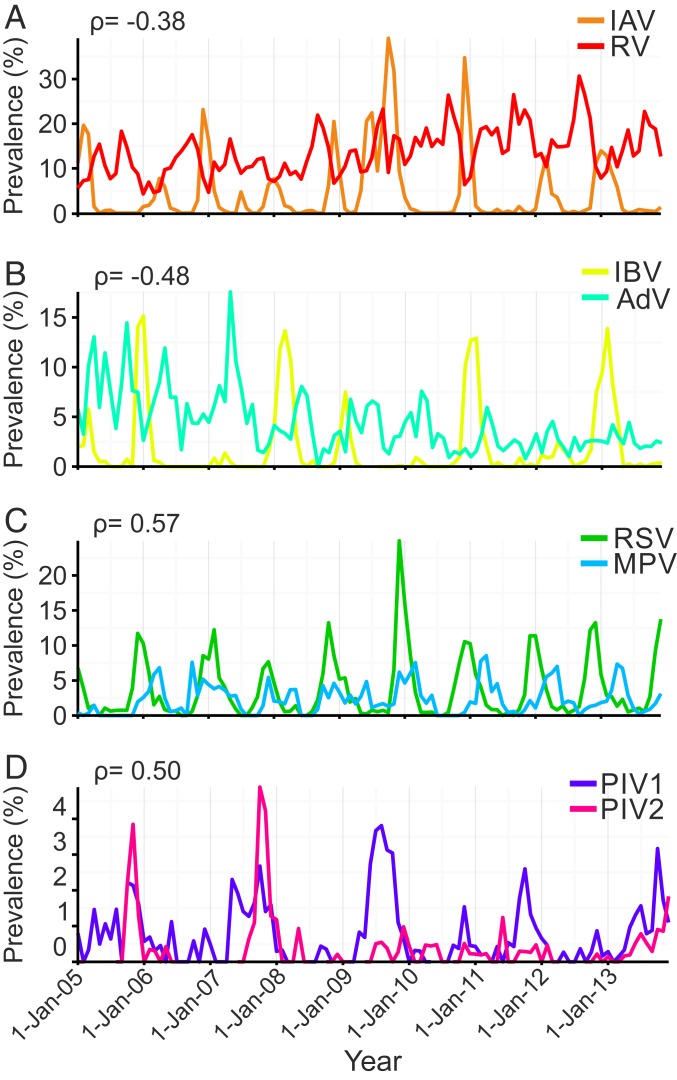
Comparative prevalences of viral infections detected among patients in Glasgow, United Kingdom, 2005 to 2013. Prevalence was measured as the proportion of patients testing positive to a given virus among those tested in each month. (*A* and *B*) Asynchronous seasonality, explained by negative epidemiological interactions. (*C* and *D*) Synchronous seasonality, explained by positive epidemiological interactions. ρ = significant correlation coefficients from Bayesian multivariate disease mapping analysis of viral infection time series shown in Fig. 3. See Table 1 for a full description of the viruses.

We first evaluated the total monthly infection prevalences across all viral respiratory infections from 2005 to 2013. As typically observed in temperate regions, the proportion of patients with respiratory illness testing positive to at least one respiratory virus peaked during winter, with the exception of the influenza A H1N1 pandemic in the summer of 2009 (Fig. 1*A*). Nevertheless, even during the influenza pandemic, the overall viral infection prevalence among patients remained broadly stable due to a simultaneous decline in the contribution of noninfluenza viruses to the total infection burden (Fig. 1*B*). Throughout the 9-y study period, because of seasonal fluctuations in the magnitude and timing of peaks in prevalences of individual viruses (Fig. 2), the dominating virus varied on a month-by-month basis (Fig. 1*B*).

### Respiratory Viruses Exhibit Cross-Correlations at the Population Level That Are Independent of Seasonality.

**Fig. 3.**
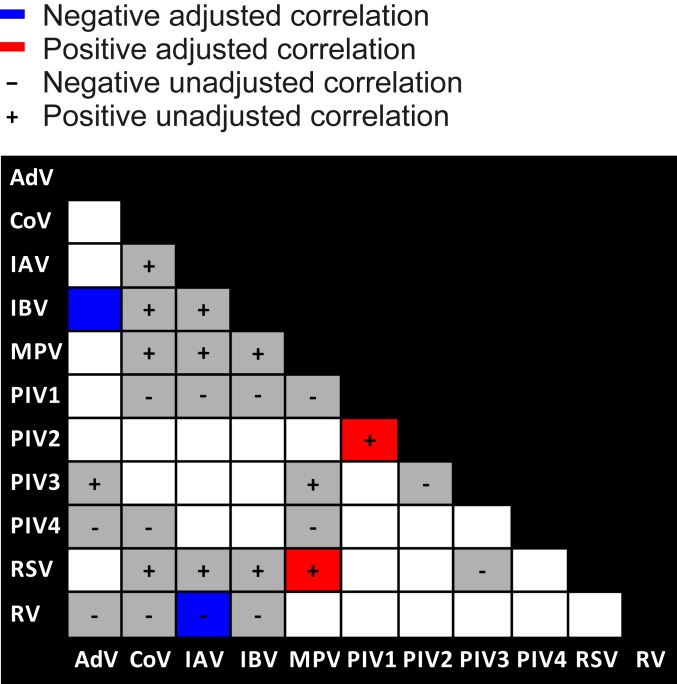
Negative and positive interactions among influenza and noninfluenza viruses at population scale. Significant unadjusted correlations from bivariate cross-correlation analysis applying Spearman’s rank method to monthly viral infection prevalences are shown in gray, with negative and positive correlations indicated by − and +, respectively, and noncorrelated virus pairs in white. Significant support for virus–virus interactions based on correlations derived from Bayesian disease mapping analysis adjusting for fluctuations in testing frequency, temporal autocorrelation, and alternative drivers of correlated seasonality are shown in blue (negative) and red (positive).

We evaluated correlations in the monthly prevalence time series for each pair of respiratory viruses. We first employed a simple bivariate nonparametric cross-correlation analysis by estimating Spearman’s rank correlation coefficients and observed 26 significant virus–virus correlations, 13 negative and 13 positive (Fig. 3, squares containing − or +, respectively). The estimated cross-correlations fall outside the 2.5% and 97.5% quantile intervals of correlation distributions generated under the null hypothesis of no interaction (see *SI Appendix*, Tables S1 and S2 and *Methods* for details). However, examining whether these are genuine virus–virus interactions (mediated at either the host or population level) required us to address several methodological limitations in this relatively simple approach: It fails to account for autocorrelation in the time series of individual viruses, or for potentially confounding factors which might independently explain correlations, and it can produce spurious negative correlations with proportional data or, alternatively, spurious positive correlations with absolute infection counts.

Traditional analytical methods are unable to address all of these limitations simultaneously, so we developed an approach that extends a multivariate Bayesian disease-mapping framework to infer interactions between virus pairs (32). This framework estimates pairwise correlations by modeling observed monthly virus counts relative to what would be expected in each month. Patient covariates age, gender, and general practice versus hospital origin (as a proxy for illness severity) were used to estimate expected counts within each month for each virus independently, capturing age and typical seasonal variability in infection risk. For example, viral exposure events may be seasonally (anti-) correlated due to similarities (differences) in the climatic preferences of viruses (25, 26), and, in some cases, due to age-dependent contact patterns driven by extensive mixing of children in daycare centers and schools (27, 28). The remaining unexplained variation includes temporal autocorrelations and dependencies between viruses. Modeling temporal autocorrelation through a hierarchical autoregressive model (32), we were able to directly estimate the between-virus correlation matrix adjusted for other key alternative drivers of infection.

This bespoke approach revealed many fewer statistically supported epidemiological interactions, with negative interactions between IAV and RV and between influenza B virus (IBV) and adenovirus (AdV) (Fig. 3, blue squares), as well as positive interactions between RSV and MPV and between PIV1 and PIV2 (Fig. 3, red squares) (*SI Appendix*, Tables S3 and S4). These interactions can be seen empirically as asynchronous (Fig. 2 *A* and *B*) and synchronous (Fig. 2 *C* and *D*) prevalence trends in the raw data. We did not detect epidemiological interactions among other possible virus pairs. We note the lack of interaction between the genetically related but antigenically dissimilar pair IAV/IBV, in line with inconsistent patterns of infection asynchrony over the 9-y time frame of our study (*SI Appendix*, Fig. S1). See *Methods* for further details.

### Within-Host Virus Mixing Patterns Are Nonrandomly Distributed across the Patient Population, Indicating Virus–Virus Interactions Operate at the Scale of Individual Hosts.

To infer virus–virus interactions at the level of individual hosts, we applied multivariable binary logistic regression to the diagnostic records of virus-positive patients. We designed our analysis to eliminate the influence of Berkson’s bias, which can lead to spuriously large or small odds ratios (ORs) when inferring disease–disease associations from hospital-based case-control data (33). To account for any influence of this potential selection bias, we restricted our analysis to the virus-positive patient subset (see *Methods* for further details).

We infer signatures of virus–virus interactions from the nonrandom patterns of virus mixing captured by coinfection information by assessing whether the propensity of a given virus X to coinfect with another virus Y was higher, lower, or equal to the overall propensity of any (remaining) virus group to coinfect with virus Y. We adjusted for the effects of age, gender, patient origin (hospital versus general practice), and the time period (with respect to the 3 major waves of the 2009 IAV pandemic). To distinguish interactions between explanatory and response viruses from unrelated seasonal changes in infection risk, we also adjusted for the monthly background prevalence of response virus infections.

**Fig. 4.**
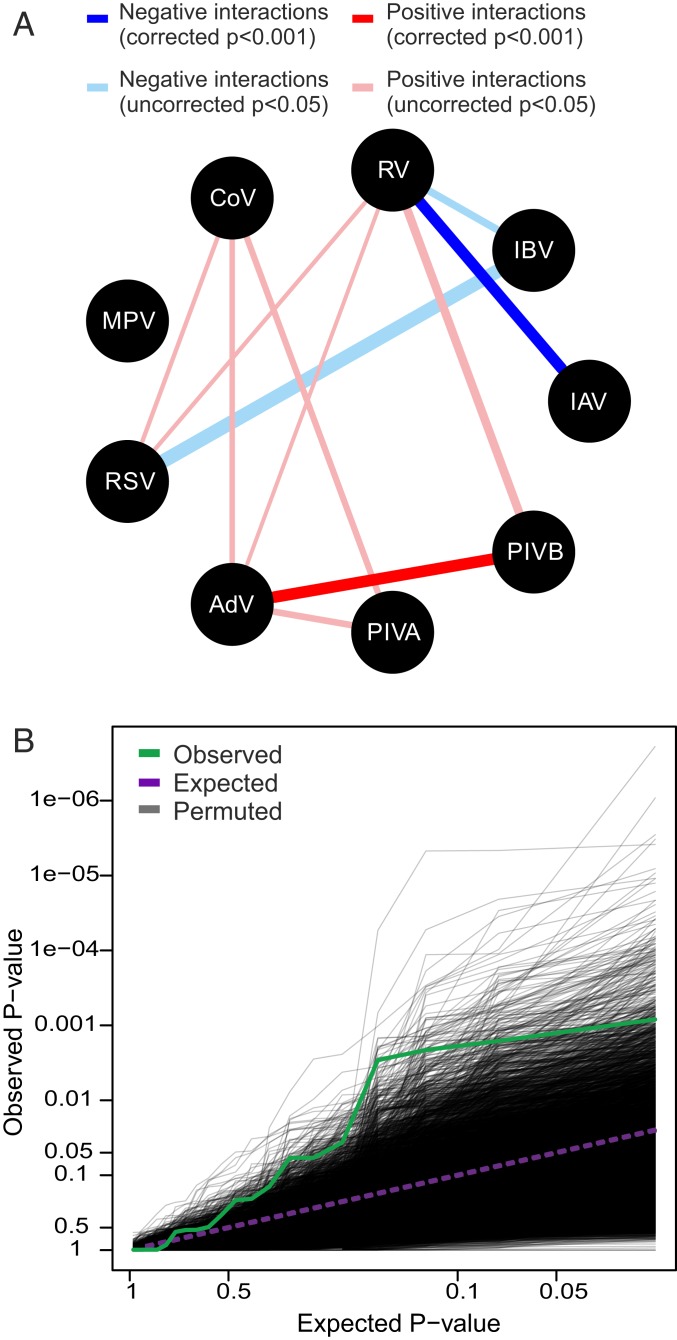
Host-scale interactions among influenza and noninfluenza viruses. (*A*) Statistically supported negative (OR < 1) and positive (OR > 1) virus–virus interactions based on uncorrected *P* < 0.05 from multivariable logistic regression analysis. Line widths are proportional to the absolute value of the maximum log OR estimated per virus pair. Two interactions (RV/IAV and AdV/PIVB) retained strong statistical support (*P* < 0.001) following Holm’s correction to control the familywise error rate. (*B*) Test of the global null hypothesis: QQ plot of the observed *P* value distribution from 20 pairwise tests among the 5 remaining virus groups (IBV, CoV, MPV, RSV, and PIVA; green line), compared to the *P* value distribution expected under the global null hypothesis of no interactions (purple dashed line). The distribution of QQ lines simulated from the global null hypothesis using 10,000 permutations is shown in gray. See Table 1 for a full description of the viruses. Due to comparatively low infection frequencies, parainfluenza viruses were regrouped into PIVA (PIV1 and PIV3; human respiroviruses) and PIVB (PIV2 and PIV4; human rubulaviruses).

As our data did not allow us to infer the directionality of virus–virus interactions, and nor did we have an a priori basis to inform this, we first performed 72 statistical tests to evaluate all 36 virus-pair hypotheses in 9 virus models (IAV, IBV, RV, RSV, human coronaviruses [CoV], AdV, MPV, PIVA [PIV1 and PIV3], and PIVB [PIV2 and PIV4]; see Table 1 for details). Due to comparatively low infection frequencies, PIVs were regrouped into PIVA (human respiroviruses) and PIVB (human rubulaviruses). Of the 72 pairwise tests, 17 yielded ORs with *P* < 0.05 before correction for multiple testing: 4 negative interactions (OR < 1) among 3 virus pairs involving influenza and 13 positive interactions (OR > 1) among 8 pairs of noninfluenza viruses (Fig. 4*A*) (note that not all significant interactions were bidirectional). We then applied Holm’s method (34) to control the familywise error rate. This confirmed the existence of 2 within-host interactions: a negative interaction between IAV and RV (OR = 0.27, *P* < 0.001) and a positive interaction between AdV and PIVB (OR = 3.99, *P* < 0.001). See *SI Appendix*, Tables S15–S17 and *Methods* for details.

**Table 1.** Details of virus detections by multiplex real-time RT-PCR assays

Abbreviation	Virus nomenclature according to the International Committee on Taxonomy of Viruses
RV	Rhinoviruses (A–C)
IAV	Influenza A virus*
IBV	Influenza B virus
RSV	(Formerly) respiratory syncytial virus^†^
CoV	Human coronaviruses (229E, NL63, HKU1)
AdV	(Formerly) human adenoviruses^‡^
MPV	Human metapneumovirus
PIV3	(Formerly) human parainfluenza 3 virus^§^
PIV1	(Formerly) human parainfluenza 1 virus^§^
PIV4	(Formerly) human parainfluenza 4 virus^¶^
PIV2	(Formerly) human parainfluenza 2 virus^¶^

* A generic assay detecting seasonal H3N2 and H1N1 subtypes and one specific to A(H1N1)pdm09.

^†^ (Currently) human orthopneumovirus.

^‡^ (Currently) human mastadenoviruses (A–G).

^§^ (Currently) human respiroviruses (1 and 3).

^¶^ (Currently) human rubulaviruses (2 and 4).

We also used a permutation method to test the global null hypothesis that there were no interactions among any of the remaining 5 virus groups (IBV, CoV, MPV, RSV, and PIVA). The distribution of *P* values tended significantly toward zero, providing strong evidence for the existence of further interactions (*P* = 0.0021; Fig. 4*B*). This indication of the likely existence of additional virus–virus interactions, that lie beyond the power permitted by our data, is consistent with the large number of candidate interactions (17 virus pairs in total) that yielded *P* < 0.05. Only 3 interactions (95% CI = 0 to 8) were expected when applying the permutation test of the global null hypothesis to all 72 tests. See *SI Appendix*, Figs. S2 and S3 and *Methods* for further details.

### Transient Immune-Mediated Cross-Protection Can Generate Linked Asynchronous Transmission Dynamics of Influenza and RV.

Our statistical analyses provide strong support for a negative interaction between seasonal IAV and the relatively ubiquitous RV, at both population and individual host scales. This negative interaction may be driven by virus competition for susceptible cells, for example as a consequence of influenza-induced destruction of cell-surface receptors (35) and/or cell death (36), or as a consequence of virus-induced innate immune responses, such as the secretion of interferon (IFN), which can cause noninfected neighboring cells to adopt a protective antiviral state (23, 24). Such biological mechanisms would render the host resistant, or only partially susceptible, to subsequent viral infection. This prompted us to ask whether a short-lived, host-scale phenomenon could explain the prominent declines in the prevalence of RV among the patient population during peak influenza activity (Fig. 2*A*).

**Fig. 5.**
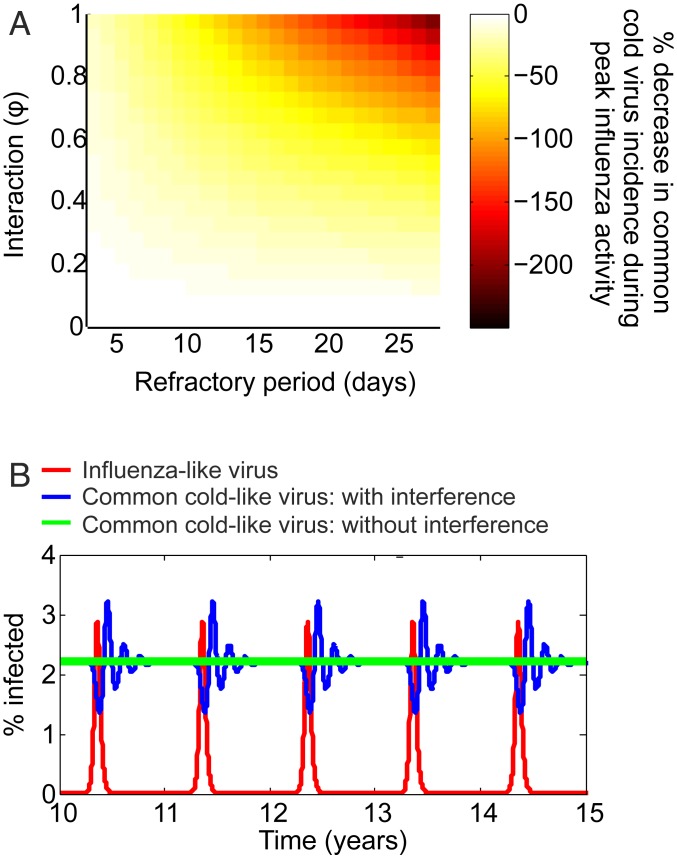
Mathematical ODE models simulating the impact of viral interference on the cocirculatory dynamics of a seasonal influenza-like virus and a ubiquitous common cold-like virus. (*A*) Percentage decrease in the minimum daily incidence of common cold-like virus infections during peak influenza-like virus activity for varying interaction strengths and refractory periods. (*B*) Asynchronous incidences of influenza-like virus (in red) and common cold-like infections in the presence (blue) and absence (green) of interference with the influenza like virus. This example assumes a strong interaction (φ = 1) and 7-d refractory period shown over 10 simulated years. The R_0_s of these viruses assuming a completely susceptible homogeneous population are 1.6 (virus 1) and 2 (virus 2). The model supports the hypothesis that temporary nonspecific protection elicited by influenza explains the periodic decline in rhinovirus frequency during peak influenza activity (Fig. 2*A*).

To address this question, we performed epidemiological simulations of the cocirculatory transmission dynamics of a seasonal influenza-like virus, such as IAV, and a nonseasonal common cold-like virus, such as RV, using ordinary differential equation (ODE) mathematical modeling (see *SI Appendix*, Fig. S4 and Table S18 and *Methods* for details). Our simulations show that a temporary reduction in host susceptibility to a secondary common cold-like virus infection, a “refractory period,” caused by a primary influenza-like virus infection is sufficient to produce an observable epidemiological impact. For example, a refractory period of just 2 d caused a 23% decrease in common cold-like virus incidence during peak influenza-like virus activity, while a refractory period of 7 d generated as much as a 61% decrease in common cold-like virus incidence (Fig. 5*A*). Notably, these simulations produced asynchronous temporal patterns of infection qualitatively similar to our empirical observations, such that the periodic decline in common cold-like virus infections coincides with peak influenza-like virus activity (Fig. 5*B*).

## Discussion

In this study, we demonstrated the presence, and examined the nature, of virus–virus interactions at both the epidemiological and individual host level by examining the diagnostic results of patients that were simultaneously tested for 11 respiratory viruses. Our study provides the most comprehensive quantitative support to date for the existence of interactions among taxonomically broad groups of respiratory viruses, building on earlier work on pathogen–pathogen interactions in the context of wildlife (37), childhood diseases (1, 38), and community ecology (39, 40). We reveal statistical support for the existence of both positive and negative interspecific interactions among respiratory viruses at both population and individual host scales. Our findings extend the established paradigms that have focused on bacteria–bacteria and bacteria–virus relationships (5) to the virus–virus case and demonstrate that respiratory virus interactions are not only restricted to previously described competitive interference forms (21).

By studying the coinfection patterns of individual patients, our analyses support an interference between influenza and noninfluenza viruses operating at the host scale. This finding supports the potential role of innate immunity, such as through the antiviral action of IFN, as early discussions on virus–virus interactions had postulated (25, 41). Capturing this potentially immune-mediated interference in mathematical simulations representing the cocirculation of a seasonal influenza-like virus and a ubiquitous common cold-like virus, we demonstrated that a short-lived protective effect, such as that induced by IFN (25), is sufficient to induce the observed asynchronous seasonal patterns we observe for IAV and RV (Fig. 2*A*). Many factors could contribute to interferences observed at the population scale through the removal of susceptible hosts (1, 38). Such effects will likely act on a timescale (on the order of days to weeks) that is similar to our proposed biological mechanism and might therefore act alternatively or in tandem to generate epidemiological interactions.

We detected a further negative interaction at the population level between IBV/AdV which was not corroborated at the individual host scale. While IBV has a (albeit inconsistent) seasonal pattern, typically peaking in winter months, AdV typically peaks around May. However, because our Bayesian hierarchical model adjusts for virus seasonality on a month-by-month basis, it is not seasonal differences that explain the negative relationship between this virus pair. In the absence of a seasonal driver or a host-scale mechanism, it is possible that the lack of cooccurrence of IBV and AdV is explained by other ecological drivers. For example, convalescence or hospitalization induced by one virus may reduce the susceptible pool at risk of exposure to other viruses, as previously discussed by others in the context of childhood diseases (1, 38).

Both IAV and IBV viruses exhibited only negative interactions at both host and population levels, although the specifics differed. That they differ in their exact pairwise interactions is unsurprising when considering that these viruses are antigenically distinct, constitute different taxonomical genera, and exhibit different viral evolutionary rates (20, 42), as well as differences in their respective age distributions of infection and some aspects of clinical presentation (43, 44). Furthermore, these viruses exhibit inconsistent patterns of cocirculation (45–47) (see also *SI Appendix*, Fig. S1) and thus their cooccurrence with other respiratory viruses is expected to vary. Based on these differences between IAV and IBV, it is feasible that their ecological relationships with other viruses have evolved differently.

Of further note is the lack of interaction detected between IAV and IBV, since there is some suggestion from global data of a short lag between their outbreak peaks. However, epidemiological data are inconsistent in that they report both asynchrony and codominance (46, 47). We believe that a lack of confirmation of interference between IAV and IBV is consistent with current virological understanding. These viruses are genetically and antigenically different; although studies have identified cross-reactive B cell and T cell epitopes in conserved regions of the viral genome (48), sufficient natural cross-protection has not been demonstrated (48, 49). It is, however, possible that their ecological relationship depends on the particular strains cocirculating. Further work is needed to better understand how the existence and nature of virus–virus interactions varies at the level of virus strains. On the other hand, some evidence exists in support of immune-driven interference between H1N1 and H3N2 subtypes of influenza A (46, 47). Our data did not permit reliable analysis at this level of virus differentiation because low and inconsistent numbers of influenza cases were routinely subtyped.

Age patterns of infection may provide important insights into the opportunity for, or alternatively the consequences of, virus–virus interactions. A lag in epidemic peaks across children and adults has been observed in the case of RSV (50, 51). Such a lag between ages may influence the potential for interaction with other cocirculating viruses, or it may reflect niche segregation as a consequence of viral interference. Although an interference between RSV and IAV has been proposed (9, 11, 48), a hypothesis recently supported in an experimental ferret model (21), this was not supported by our data. It is possible that such an effect was masked because our analysis did not enable stratification of effects by age, a step that is anticipated to severely limit the statistical power of our highly comprehensive analysis of virus–virus interactions. More detailed investigation to address more precisely when, and among whom, virus–virus interactions occur will be an important challenge for future research.

Our study describes positive interactions among respiratory viruses at the population scale. These positive epidemiological interactions were not mirrored at the host scale, which suggests they are independent of host-scale factors and may instead be explained by variables that were not captured by our study. For example, some respiratory viruses, such as RSV and MPV, are known to enhance the incidence of pneumococcal pneumonia (6, 52). It is possible that we are observing an intermediary effect of secondary bacterial infections, which may lead to enhanced hospitalization with both viruses, rather than a direct virus–virus interaction.

Although not mirrored at the population level, our 72 pairwise tests did detect the existence of positive interactions among 8 pairs of viruses, including strong statistical support for AdV/PIVB following Holm’s correction for multiple comparisons. This finding is consistent with a recent, smaller-scale clinical study of children diagnosed with pneumonia, which detected 2 pairs of positively associated noninfluenza viruses (17). Plausible mechanisms of positive virus–virus interactions include enhanced viral growth through syncytia formation of a coinfecting virus (22), the virus-mediated down-regulation of IFN, which may promote invasion by opportunistic viruses (41), and the virus-induced release of cytokines such as interleukin 10, which prevents the detrimental effects of dysregulated immune responses and might lead to immunosuppression and enhanced susceptibility to secondary viral infections (53, 54). That most interactions detected at the host scale were not supported at the population level is not surprising given that interaction effects are reliant on coinfection, or sequential infections, occurring within a short time frame. The relative rareness of interaction events might thus limit their detectability and epidemiological impact.

It is important to consider that virus–virus interactions are likely shaped by evolutionary processes and thus their existence, magnitude, and dominant form (whether competitive or facilitative) may not be static in nature, and instead depend on particular virus strains, as well as host and environmental factors (55). The virus–virus interactions we detect may represent a “net effect” of the evolutionary processes (55) driving the virus strains circulating in the UK population over the 9-y timeframe of this study. It should also be borne in mind that a large proportion of respiratory infections, including influenza, are expected to be asymptomatic (56), and coinfections of some viruses may be associated with attenuated disease (23, 57). It is therefore conceivable that the form of interaction detected in a patient population, although of clinical importance, may differ from that occurring in the community at large.

Our study provides strong statistical support for the existence of interactions among genetically broad groups of respiratory viruses at both population and individual host scales. Our findings imply that the incidence of influenza infections is interlinked with the incidence of noninfluenza viral infections with implications for the improved design of disease forecasting models and the evaluation of disease control interventions. Future experimental studies are required to decipher the biological mechanisms that underpin virus–virus interactions and their effects on the within-host dynamics of infection.

## Methods

### Study Population and Dataset.

Our study was based on routine diagnostic test data used to inform the laboratory-based surveillance of acute respiratory infections in NHS Greater Glasgow and Clyde (the largest Health Board in Scotland), spanning primary, secondary, and tertiary healthcare settings. Clinical specimens were submitted to the West of Scotland Specialist Virology Centre for virological testing by multiplex real-time RT-PCR (58, 59). Patients were tested for 11 groups of respiratory viruses summarized in Table 1.

Data were available to our study for the period January 2005 to December 2013 comprising 61,427 clinical samples, most of which were nasal and/or throat swabs (98%). The test results of individual samples were aggregated to the patient level using a window of 30 d to define a single episode of illness, giving an overall infection status per episode of respiratory illness. This yielded a total of 44,230 episodes of respiratory illness from 36,157 individual patients. In this study population, 35% (15,302) of patient episodes were virus-positive, 8% (1247) of which were coinfected with more than one virus group.

These data provide a coherent source of routine laboratory-based data for inferring epidemiological patterns of respiratory illness, reflecting typical community-acquired respiratory virus infections in a large urban population (60). Of the total 44,230 episodes of illness, 62% (27,284) were tested for all 11 virus groups, increasing to 83% (22,420) among 26,974 episodes tested out with the 3 major waves of A(H1N1)pdm09 virus circulation. More than 99% of patients were tested for all viruses in each calendar month (except for AdV in 2005 to 2007, PIV4 in 2005, and during pandemic influenza). Virological diagnostic assays remained consistent over the 9-y period, with the exception of the RV assay, which was modified during 2009 to detect a wider array of RV and enteroviruses (including D68), and 1 of 4 CoV assays (CoV-HKU1) was discontinued in 2012.

These diagnostic data included test-negative results providing the necessary denominator data to account for fluctuations in testing frequencies across patient groups and over time. Such changes in testing patterns may be expected to arise in healthcare data accrued over extended time frames, for example because of changes in healthcare-seeking behavior and/or the clinical management of patients. We refer readers to ref. 60 for further details of the dataset and epidemiological patterns of virus detections.

### Bivariate Cross-Correlation Analysis.

Spearman’s rank correlation coefficients were computed and tested between all pairs of virus infection prevalences (the proportion positive among those tested) in each month using cor.test in R (32) version 3.4.4 (61). These analyses were based on 26,974 patient episodes of respiratory illness excluding the period spanning the 3 major waves of A(H1N1)pdm09 virus circulation. We additionally conducted an analysis of the distribution of correlation coefficients generated under the null hypothesis of no virus–virus interactions. To do so, we randomly permuted the monthly prevalence time series of each virus pair 1,000 times and computed the 2.5% and 97.5% quantiles of each distribution of correlation coefficients. See *SI Appendix*, Tables S1 and S2 for the estimated correlation coefficients, distributions under the null hypothesis, and *P* values.

### Multivariate Bayesian Hierarchical Models.

Although the simple bivariate cross-correlation analysis revealed several significantly correlated virus pairs, inferring genuine virus–virus interactions using diagnostic patient data requires several features to be accounted for: Absolute infection counts must be adjusted for fluctuations in testing frequency, temporal autocorrelation, and alternative confounding explanations of temporal dependency between virus groups.

To address these methodological limitations, we developed and applied a statistical approach that extends a multivariate Bayesian hierarchical modeling method to times-series data (32). The method employs Poisson regression to model observed monthly infection counts adjusting for confounding covariates and underlying test frequencies. This framework uses a multivariate normal distribution to model the random temporal variation in the adjusted virus counts, naturally incorporating a between-virus covariance matrix that enabled estimation of virus–virus correlations. Through estimating, and scaling, the off-diagonal entries of this matrix, we were able to estimate posterior interval estimates for correlations between each virus pair. Under a Bayesian framework, posterior probabilities were estimated to assess the probability of zero being included in each interval (one for each virus pair). Adjusting for multiple comparisons, correlations corresponding to intervals with an adjusted probability less than 0.05 were deemed significantly different from zero. Crucially, the method makes use of multiple years of data, allowing expected annual patterns for any virus to be estimated, thereby accounting for typical seasonal variability in infection risk while also accounting for covariates such as patient age (as well as gender and hospital vs. general practice [GP] patient origin). We identified significant interactions between IAV/RV, IBV/AdV, RSV/MPV, and PIV1/PIV2. See *SI Appendix*, Tables S3 and S4 for the pairwise correlation estimates summarized in Fig. 3 of the main text, and see ref. 32 for further details on the method itself.

### Host-Scale Analyses: Binary Logistic Regression.

We analyzed the patient-level coinfection data with a series of binary logistic regression models and evaluate whether virus–virus interactions may be explained by interactions operating at the scale of individual hosts. Of the 44,230 total episodes of respiratory illness, 73% represent patients experiencing a single episode during the 9-y study period, while the remaining 27% represent patients experiencing 2 or more episodes (range 2 to 26; mean = 3 episodes per patient). Retaining the first observed episodes generated 36,157 patient records and 20,703 with complete testing across all virus groups [note that pressures on laboratory resources led to high partial testing during the major waves of A(H1N1)pdm09 virus circulation]. Of these 20,703 patients, 6,884 were virus-positive, 11% of which were coinfected with 2 or more viruses. We note that the coinfection prevalence and detected virus–virus interactions were robust to the choice of window used to define an episode of illness when aggregating individual samples (see *SI Appendix*, Table S19 for details).

We designed our analysis to minimize the potential influence of Berkson’s bias—a form of selection bias arising in studies aiming to infer disease–disease associations from hospital-based case-control data. This bias arises where there is an underlying difference in the probabilities of study inclusion between case and control groups (33). Our approach was, for each study virus (Y) in turn, to restrict the analysis to patients positive for one of the other (explanatory) viruses, thereby eliminating the influence of the underrepresentation of the virus-negative “healthy” population, implicit in the patient-based study population. The study population comprised individuals infected with at least one other (non-Y) virus. Within that group, exposed individuals were positive to virus X, and unexposed individuals were negative to virus X. Cases were coinfected with virus Y, while controls were negative to virus Y. In this way, our analysis quantifies whether the propensity of virus X to coinfect with virus Y was more, less, or equal to the overall propensity of any (remaining) virus group to coinfect with Y. Our study therefore infers signatures of virus–virus interactions from the nonrandom patterns of virus mixing among the virus-positive population.

Our analyses adjusted for key predictors of respiratory virus infections: patient age (AGE.CAT), patient sex (SEX), hospital vs. GP patient origin (ORIGIN), and time period of sample collection with respect to the influenza A(H1N1)pdm09 virus pandemic (PANDEMIC). In addition, we ensured that virus–virus associations were not simply explained by independent monthly fluctuations in the response virus infection risk. To do so, we adjusted the total number of infections with the response virus (VCOUNT) and the total number tested (TCOUNT) within a 15-d window either side of each (earliest) sample collection date for each individual observation. Model variables were either binary or categorized; see *SI Appendix*, Table S5 for details.

Specifically, the relative odds of coinfection with virus Y (versus any other virus group) was estimated for each of the 8 explanatory viruses, for each response virus Y. Thus, each virus model (IAV, IBV, RV, RSV, CoV, AdV, MPV, PIVA, and PIVB) used a specific subset of the virus-positive population (because each virus model excluded virus-negative patients and single infections involving only the response virus Y) with each data subset ranging from 4,629 to 6,743 (see *SI Appendix*, Tables S6–S14 for details). Due to low infection frequencies, we regrouped PIVs into 2 groups: PIVA (PIV1 and PIV3; human respiroviruses) and PIVB (PIV2 and PIV4; human rubulaviruses).

Each of the 9 virus models followed the following form, where $P_{i}^{Y}$ indicates the probability that individual *i* is coinfected with virus Y, and *X*1…*X*8 indicates the 8 explanatory viruses:

$$\begin{array}{l} log\left(\frac{P_{i}^{Y}}{1-P_{i}^{Y}}\right)=\beta_{0}+\beta_{1}AGE.CAT_{i}+\beta_{2}SEX_{i}+\beta_{3}ORIGIN_{i}+\beta_{4}PANDEMIC_{i}\\ +\beta_{5}\log\left(V.COUNT_{i}+1\right)+\beta_{6}\log\left(T.COUNT_{i}+1\right)+\beta_{7}X1_{i}+\beta_{8}X2_{i}+\beta_{9}X3_{i}\\ +\beta_{10}X4_{i}+\beta_{11}X5_{i}+\beta_{12}X6_{i}+\beta_{13}X7_{i}+\beta_{14}X8_{i}. \end{array}$$

Analyses were conducted using the glm function in R version 3.4.4 (61). The quality of each model was assessed by the predictive power given by the area under the receiver operator characteristic curve. Because these data do not allow for inferences of the directionality of the virus–virus interactions, we conducted all possible 72 pairwise statistical tests in the first instance to evaluate 36 virus-pair hypotheses and present results where ORs yield unadjusted *P* < 0.05. These analyses detected 17 significant virus–virus interaction (4 negative and 13 positive) out of the 72 tests (*SI Appendix*, Tables S15 and S16). Not all virus-pair interactions were bidirectional; however, we note that these analyses do not allow the differentiation of cause from effect.

Applying Holm’s method to control the probability of one or more false discoveries arising among the family of 72 tests [using the p.adjust function in R version 3.4.4 (61)] yielded strong evidence (*P* < 0.001 following correction) of the existence of both negative (IAV/RV) and positive (AdV/PIVB) interactions (see *SI Appendix*, Table S17 for details). A permutation test of the global null hypothesis was then applied to the 5 remaining virus groups (IBV, CoV, MPV, RSV, and PIVA) to test the hypothesis that the 20 remaining null hypotheses tested were true. Using Fisher’s method of combining *P* values (62), we found strong evidence of association among these 5 virus groups (*P* = 0.0021; *SI Appendix*, Fig. S2), although we expect nonindependence between these tests. We therefore accounted for nonindependence among the pairwise tests by using permutations to simulate the null distribution of combined *P* values. Each generalized linear model was fitted to 10,000 datasets where the null hypothesis was simulated by permuting the response variable (virus Y). The significant tendency of the distribution of *P* values toward zero (and away from the uniform distribution expected under the null hypothesis) was illustrated using a quantile–quantile (QQ) plot to compare the 20 observed *P* values with the 10,000 distributions simulated under the global null hypothesis (Fig. 3*C*). The signal of additional interactions was further demonstrated when the permutation test of the global null hypothesis was extended to all 72 tests (*SI Appendix*, Fig. S3)—only 3 (95% CI: 0 to 9) interactions were expected by chance assuming the null hypothesis of no interactions, in comparison to the 17 interactions we detected.

### Mathematical Modeling of Influenza and RV Interactions and Population Impact.

We developed a 2-pathogen deterministic SIR-type mechanistic model to study the population dynamics of a seasonal influenza-like virus and a ubiquitous common cold-like virus cocirculation. We used this framework to compare the frequency of common cold-like virus infections with and without an interference with the influenza-like virus. A schematic representation of the model is provided in *SI Appendix*, Fig. S4. The temporal dynamics of the viruses were distinguished in 2 key ways. First, seasonal forcing was applied to the influenza-like virus (virus 1) via a sinusoidally varying transmission rate. Second, the rate of waning immunity of the common cold-like virus (virus 2) was assumed to be 10 times faster than for the influenza-like virus. This more rapid replenishment of susceptible individuals was designed to reflect the high year-round prevalence and diversity of circulating subtypes that are characteristic of RV infections (63).

Individuals were assumed to acquire primary infections at rate β (days^−1^), assuming frequency-dependent transmission, and subsequently transition from susceptible (S) to an infectious refractory phase (I). Infected individuals were assumed not to be susceptible to further infections with the primary infecting virus. Our assumption is that multiple exposures to similar virus strains are unlikely to alter the within-host dynamics during this short period. At the end of the infectious period, individuals entered a noninfectious refractory phase (J) at rate 1/γ (days^−1^). This second refractory phase was designed to reflect immune effects that may persist for a period beyond viral clearance (64, 65). During both refractory phases, viral interactions are captured via reduced susceptibility of influenza-like virus infected individuals to either coinfection with the common cold-like virus (during phase I) or, alternatively, a secondary infection with the common cold-like virus (during phase J). The strength of the interaction was determined by an interaction parameter *φ* applied to the transmission rate, whereby *φ* = 0 induced complete susceptibility (equivalent to transmission dynamics in the absence of an interaction), 0 < *φ *< 1 induced a partial reduction in susceptibility, and *φ* = 1 induced a complete loss of susceptibility.

At the end of the second refractory phase, infected individuals developed long-lasting immunity to infection due to antibodies generated at rate *φ* (days^−1^) (R). During this phase, individuals were not susceptible to the primary infection but could acquire secondary infections if previously unexposed. Following a period of long-term immunity individuals reverted to susceptible (S) at a rate σ (days^−1^) representing the waning of antibodies against the primary infection.

The full model ODEs are given as follows:

$$\frac{\text{d}\left\{\text{SS}\right\}}{\text{dt}}=\mu +\sigma_{1}\left\{\text{RS}\right\}+\sigma_{2}\left\{\text{SR}\right\}-\beta_{1}\left(\text{t}\right)\left\{\text{SS}\right\}{\text{I}}_{1}^{'}-\beta_{2}\left\{\text{SS}\right\}{\text{I}}_{2}^{'}-\mu \left\{\text{SS}\right\}$$

$$\frac{\text{d}\left\{\text{IS}\right\}}{\text{dt}}=\beta_{1}\left(\text{t}\right)\left\{\text{SS}\right\}{\text{I}}_{1}^{'}+\sigma_{2}\left\{\text{IR}\right\}-\gamma_{1}\left\{\text{IS}\right\}-\beta_{2}\left(1-\varphi \right)\left\{\text{IS}\right\}{\text{I}}_{2}^{'}-\mu \left\{\text{IS}\right\}$$

$$\frac{\text{d}\left\{\text{JS}\right\}}{\text{dt}}=\gamma_{1}\left\{\text{IS}\right\}+\sigma_{2}\left\{\text{JR}\right\}-\omega_{1}\left\{\text{JS}\right\}-\beta_{2}\left(1-\varphi \right)\left\{\text{JS}\right\}{\text{I}}_{2}^{'}-\mu \left\{\text{JS}\right\}$$

$$\frac{\text{d}\left\{\text{RS}\right\}}{\text{dt}}=\omega_{1}\left\{\text{JS}\right\}+\sigma_{2}\left\{\text{RR}\right\}-\sigma_{1}\left\{\text{RS}\right\}-\beta_{2}\left\{\text{RS}\right\}{\text{I}}_{2}^{'}-\mu \left\{\text{RS}\right\}$$

$$\frac{\text{d}\left\{\text{SI}\right\}}{\text{dt}}=\sigma_{1}\left\{\text{RI}\right\}+\beta_{2}\left\{\text{SS}\right\}{\text{I}}_{2}^{'}-\beta_{1}\left(\text{t}\right)\left\{\text{SI}\right\}{\text{I}}_{1}^{'}-\gamma_{2}\left\{\text{SI}\right\}-\mu \left\{\text{SI}\right\}$$

$$\frac{\text{d}\left\{\text{II}\right\}}{\text{dt}}=\beta_{1}\left(\text{t}\right)\left\{\text{SI}\right\}{\text{I}}_{1}^{'}+\beta_{2}\left(1-\varphi \right)\left\{\text{IS}\right\}{\text{I}}_{2}^{'}-\gamma_{1}\left\{\text{II}\right\}-\gamma_{2}\left\{\text{II}\right\}-\mu \left\{\text{II}\right\}$$

$$\frac{\text{d}\left\{\text{JI}\right\}}{\text{dt}}=\gamma_{1}\left\{\text{II}\right\}+\beta_{2}\left(1-\varphi \right)\left\{\text{JS}\right\}{\text{I}}_{2}^{'}-\omega_{1}\left\{\text{JI}\right\}-\gamma_{2}\left\{\text{JI}\right\}-\mu \left\{\text{JI}\right\}$$

$$\frac{\text{d}\left\{\text{RI}\right\}}{\text{dt}}=\omega_{1}\left\{\text{JI}\right\}+\beta_{2}\left\{\text{RS}\right\}{\text{I}}_{2}^{'}-\sigma_{1}\left\{\text{RI}\right\}-\gamma_{2}\left\{\text{RI}\right\}-\mu \left\{\text{RI}\right\}$$

$$\frac{\text{d}\left\{\text{SR}\right\}}{\text{dt}}=\sigma_{1}\left\{\text{RR}\right\}+\gamma_{2}\left\{\text{SI}\right\}-\beta_{1}\left(\text{t}\right)\left\{\text{SR}\right\}{\text{I}}_{1}^{'}-\sigma_{2}\left\{\text{SR}\right\}-\mu \left\{\text{SR}\right\}$$

$$\frac{\text{d}\left\{\text{IR}\right\}}{\text{dt}}=\beta_{1}\left(\text{t}\right)\left\{\text{SR}\right\}{\text{I}}_{1}^{'}+\gamma_{2}\left\{\text{II}\right\}-\gamma_{1}\left\{\text{IR}\right\}-\sigma_{2}\left\{\text{IR}\right\}-\mu \left\{\text{IR}\right\}$$

$$\frac{\text{d}\left\{\text{JR}\right\}}{\text{dt}}=\gamma_{1}\left\{\text{IR}\right\}+\gamma_{2}\left\{\text{JI}\right\}-\omega_{1}\left\{\text{JR}\right\}-\sigma_{2}\left\{\text{JR}\right\}-\mu \left\{\text{JR}\right\}$$

$$\frac{\text{d}\left\{\text{RR}\right\}}{\text{dt}}=\omega_{1}\left\{\text{JR}\right\}+\gamma_{2}\left\{\text{RI}\right\}-\sigma_{1}\left\{\text{RR}\right\}-\sigma_{2}\left\{\text{RR}\right\}-\mu \left\{\text{RR}\right\}$$

$$\beta_{1}\left(\text{t}\right)=\text{B*}\left(1+\text{b*}\sin\left(2\text{*π*}\frac{\text{t}}{365}\right)\right)$$

$${\text{I}}_{1}^{'}=\text{IS}+\text{II}+\text{IR}$$

$${\text{I}}_{2}^{'}=\text{SI}+\text{II}+\text{JI}+\text{RI},$$

where {1,2} denotes the infection status of individuals with respect to virus 1 (the seasonal influenza-like virus) and virus 2 (the nonseasonal common cold-like virus) for each of 12 combinations of infection classes, whereby S = susceptible, I = infectious refractory phase, J = noninfectious refractory phase, R = immunity phase, and subscripts 1 and 2 denote the corresponding virus-specific parameters. For example, {SI} indicates the state of being susceptible to virus 1 and infectious with virus 2. ${\text{I}}_{1}^{'}$ and ${\text{I}}_{2}^{'}$ represent the total proportion of individuals infectious with virus 1 and virus 2 respectively; β_1_(t) denotes the time-dependent rate at which new infections with virus 1 are generated and comprises a fixed transmission parameter B together with a seasonal amplitude parameter b; and *φ* represents a background, nonacute-respiratory-infection–induced rate of mortality given by 1/the average life expectancy (days^−1^). The peak proportion of individuals coinfected with both viruses was 0.39% during each simulated season of influenza-like virus circulation. The R_0_s of these 2 viruses assuming a completely susceptible homogeneous population are 1.6 (virus 1) and 2 (virus 2). Full parameter values and ranges are provided in *SI Appendix*, Table S18.

This framework was implemented in MATLAB software v.R2013b using the ode45 differential equation solver. Each simulation was run for a period of 20 y; a “burn-in” period of 10 y was excluded from the analyses. Using this framework, we quantified the effect of transient immune-mediated viral interactions on the percentage decrease in daily nonseasonal common cold-like virus prevalence during peak seasonal influenza-like virus activity.

### Data Availability.

The patient-level data used in this study are available upon request to NHS Scotland (https://www.informationgovernance.scot.nhs.uk/pbpphsc/home/for-applicants/). Aggregated forms of summary data and computer code may be made available upon request to the corresponding author.

## Supplementary Material

Supplementary File
