# Supplementary material for: Virus–virus interactions impact the population dynamics of influenza and the common cold
Source: Proc Natl Acad Sci U S A. 2019 Dec 16;116(52):27142–50. doi: 10.1073/pnas.1911083116 (PMC6936719; doi:10.1073/pnas.1911083116)
Supplement: Supplementary File [file pnas.1911083116.sapp.pdf]

Supporting Information Appendix for

Virus-virus interactions impact the population dynamics of influenza and the common cold

Sema Nickbakhsh, Colette Mair, Louise Matthews, Richard Reeve, Paul C.D. Johnson, Fiona Thorburn, Beatrix von Wissmann, Arlene Reynolds, James McMenamin, Rory N. Gunson, and Pablo R. Murcia

Correspondence to: Pablo.Murcia@glagow.ac.uk or Louise.Matthews@glasgow.ac.uk

www.pnas.org/cgi/doi/10.1073/pnas.1911083116

Table S1: Bivariate Spearman’s cross-correlation coefficients estimated for each pair of virus infection time series data (monthly prevalence). Significance was assessed by asymptotic (*t* approximation) p-values <0.05 (see Table S2). Red indicates significant positive coefficients, blue indicates significant negative coefficients, and gray indicates coefficients not meeting the criteria of significance. Lower (2.5%) and upper (97.5%) quantiles from Spearman’s rank coefficient distributions estimated under the null hypothesis of no virus-virus interactions are given in parentheses. The distribution under the null hypothesis was generated by randomly permuting each pair of virus infection time series data (monthly prevalence) 1000 times.

**AdV**											
**CoV**	0.065 (-0.197, 0.219)										
**IAV**	-0.002 (-0.214, 0.211)	0.616 (-0.201, 0.208)									
**IBV**	0.011 (-0.203, 0.222)	0.496 (-0.208, 0.216)	0.388 (-0.223, 0.206)								
**MPV**	-0.034 (-0.226, 0.223)	0.509 (-0.207, 0.212)	0.311 (-0.214, 0.204)	0.282 (-0.219, 0.201)							
**PIV1**	-0.029 (-0.216, 0.213)	-0.268 (-0.207, 0.234)	-0.248 (-0.203, 0.217)	-0.269 (-0.22, 0.202)	-0.230 (-0.215, 0.211)						
**PIV2**	-0.20 (-0.222, 0.211)	-0.053 (-0.221, 0.222)	-0.094 (-0.206, 0.204)	-0.038 (-0.204, 0.22)	-0.026 (-0.214, 0.199)	0.545 (-0.215, 0.214)					
**PIV3**	0.329 (-0.206, 0.207)	-0.048 (-0.217, 0.209)	-0.027 (-0.209, 0.218)	0.115 (-0.211, 0.198)	0.263 (-0.211, 0.202)	-0.204 (-0.208, 0.212)	-0.360 (-0.211, 0.22)				
**PIV4**	-0.229 (-0.228, 0.22)	-0.228 (-0.222, 0.215)	-0.197 (-0.217, 0.232)	-0.220 (-0.216, 0.232)	-0.285 (-0.22, 0.231)	0.173 (-0.224, 0.224)	0.148 (-0.222, 0.226)	-0.213 (-0.211, 0.230)			
**RSV**	-0.193 (-0.206, 0.203)	0.607 (-0.22, 0.196)	0.425 (-0.209, 0.212)	0.305 (-0.21, 0.231)	0.385 (-0.215, 0.207)	0.060 (-0.204, 0.224)	0.186 (-0.215, 0.22)	-0.308 (-0.227, 0.201)	0.008 (-0.23, 0.226)		
**RV**	-0.386 (-0.203, 0.205)	-0.456 (-0.206, 0.20)	-0.545 (-0.224, 0.229)	-0.288 (-0.227, 0.219)	-0.109 (-0.199, 0.201)	0.052 (-0.20, 0.20)	0.033 (-0.217, 0.22)	0.037 (-0.209, 0.204)	0.158 (-0.221, 0.225)	-0.203 (-0.2, 0.221)	
	**AdV**	**CoV**	**IAV**	**IBV**	**MPV**	**PIV1**	**PIV2**	**PIV3**	**PIV4**	**RSV**	**RV**

Table S2: Bivariate cross-correlation asymptotic (t approximation) p-values estimated by Spearman's rank method between each pair of virus infection time series data (monthly prevalence). Significance was based on p-value <0.05; significant positive correlations are in red, significant negative correlations are in blue, and insignificant correlations are in gray.

**AdV**											
**CoV**	0.5551										
**IAV**	0.9835	<0.001									
**IBV**	0.9193	<0.001	0.0002								
**MPV**	0.7528	<0.001	0.0036	0.0084							
**PIV1**	0.7918	0.0127	0.0213	0.0124	0.0331						
**PIV2**	0.0652	0.6249	0.3885	0.7280	0.8087	<0.001					
**PIV3**	0.0020	0.6618	0.8066	0.2897	0.0290	0.0589	0.0007				
**PIV4**	0.0452	0.0464	0.0860	0.0544	0.0119	0.1322	0.2000	0.0629			
**RSV**	0.0744	<0.001	<0.001	0.0042	0.0003	0.5806	0.0862	0.0039	0.9483		
**RV**	0.0002	<0.001	<0.001	0.0073	0.3200	0.6349	0.7614	0.7352	0.1694	0.0611	
	**AdV**	**CoV**	**IAV**	**IBV**	**MPV**	**PIV1**	**PIV2**	**PIV3**	**PIV4**	**RSV**	**RV**

Table S3: Correlation coefficients estimated multivariately using Bayesian hierarchical modelling, adjusting for fluctuations in testing frequency, autocorrelation and covariates (age, gender and general practice vs. hospital sample origin). Red indicates significant positive coefficients, blue indicates significant negative coefficients, and gray indicates coefficients not meeting the criteria of significance. Significance was indicated by a p-value <0.05 following a correction for multiple comparisons (see (1) for details) and a higher posterior density (HPD) estimate that excludes zero (see Table S5 below). Significant pairs are indicative of viral interactions and are presented in color in Fig.3 of the main text.

**AdV**											
**CoV**	0.11										
**IAV**	0.2	-0.06									
**IBV**	-0.48	0.15	-0.23								
**MPV**	-0.11	-0.48	-0.21	-0.39							
**PIV1**	0.22	-0.27	0.24	-0.31	0.19						
**PIV2**	0.16	-0.11	-0.01	-0.32	0	0.5					
**PIV3**	0.14	-0.22	-0.04	0.14	0.23	-0.03	-0.31				
**PIV4**	0.05	-0.22	0.1	0.22	0.15	0.16	-0.05	0.11			
**RSV**	-0.09	-0.01	-0.13	-0.21	0.57	0.01	0.05	0.27	0.03		
**RV**	-0.02	-0.08	-0.38	-0.22	-0.06	0.09	0.12	0.06	-0.16	0.15	
	**AdV**	**CoV**	**IAV**	**IBV**	**MPV**	**PIV1**	**PIV2**	**PIV3**	**PIV4**	**RSV**	**RV**

Table S4. Bayesian hierarchical model higher posterior density (HPD) estimates indicating the significance of correlation coefficient point estimates. Red indicates significant positive coefficients, blue indicates significant negative coefficients, and gray indicates coefficients not meeting the criteria of significance.

**AdV**											
**CoV**	(−0.26, 0.47)										
**IAV**	(−0.11, 0.53)	(−0.3, 0.23)									
**IBV**	(−0.68, −0.21)	(−0.14, 0.42)	(−0.45, −0.01)								
**MPV**	(−0.38, 0.17)	(−0.69, −0.18)	(0, 0.42)	(−0.61, −0.14)							
**PIV1**	(−0.11, 0.52)	(−0.56, 0.05)	(−0.02, 0.48)	(−0.59, 0.02)	(−0.05, 0.43)						
**PIV2**	(−0.27, 0.52)	(−0.45, 0.28)	(−0.41, 0.37)	(−0.68, 0)	(−0.34, 0.28)	(0.21, 0.73)					
**PIV3**	(−0.27, 0.47)	(−0.54, 0.11)	(−0.35, 0.26)	(−0.13, 0.43)	(−0.03, 0.46)	(−0.38, 0.32)	(−0.65, 0.13)				
**PIV4**	(−0.4, 0.46)	(−0.56, 0.24)	(−0.22, 0.52)	(−0.1, 0.57)	(−0.1, 0.41)	(−0.27, 0.54)	(−0.6, 0.43)	(−0.26, 0.45)			
**RSV**	(−0.41, 0.26)	(−0.29, 0.28)	(−0.37, 0.14)	(−0.46, 0.05)	(0.37, 0.7)	(−0.31, 0.39)	(−0.37, 0.4)	(−0.06, 0.57)	(−0.35, 0.47)		
**RV**	(−0.38, 0.31)	(−0.4, 0.22)	(−0.61, −0.13)	(−0.5, 0.07)	(−0.29, 0.16)	(−0.21, 0.41)	(−0.36, 0.49)	(−0.3, 0.37)	(−0.53, 0.27)	(−0.12, 0.42)	
	**AdV**	**CoV**	**IAV**	**IBV**	**MPV**	**PIV1**	**PIV2**	**PIV3**	**PIV4**	**RSV**	**RV**

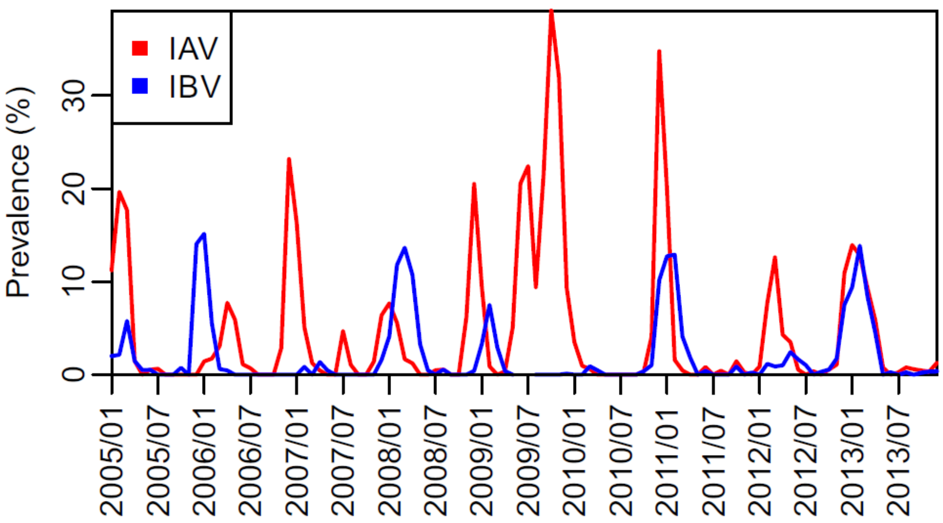

Fig. S1: Monthly infection prevalence of influenza A (IAV) and influenza B (IBV) viruses among the patient population from 2005 to 2013. IAV and IBV exhibit inconsistent patterns of co-circulation: synchrony, a lag in peak timing, and dominance of one species, are all observed over the nine-year time frame of the study.

Table S5: Distribution of explanatory factors among 6,884* respiratory virus positive patients tested for 11 virus groups in the Greater Glasgow & Clyde Health Board, 2005-2013.

Explanatory variable	Levels	No. of patients (%)
Age group (years)	1-5 (reference)	1639 (23.8)
	<1	1205 (17.5)
	6-16	703 (10.2)
	17-45	1419 (20.6)
	46-64	1094 (15.9)
	≥65	824 (12.0)
Sex	Female (reference)	3310 (48.1)
	Male	3574 (51.9)
Illness severity‡	GP (reference)	1734 (25.2)
	Hospital	4606 (66.9)
	Intensive care	544 (7.9)
Pandemic period‡	Pre-pandemic (reference)	2175 (31.6)
	Pandemic	1247 (18.1)
	Post-pandemic	3462 (50.3)
IAV infection	No (reference)	6078 (88.3)
	Yes	806 (11.7)
IBV infection	No (reference)	6314 (91.7)
	Yes	570 (8.3)
RV infection	No (reference)	4211 (61.2)
	Yes	2673 (38.8)
RSV infection	No (reference)	5972 (86.8)
	Yes	912 (13.2)
CoV infection	No (reference)	6089 (88.5)
	Yes	795 (11.5)
AdV infection	No (reference)	6219 (90.3)
	Yes	665 (9.7)
MPV infection	No (reference)	6351 (92.3)
	Yes	533 (7.7)
PIVA infection	No (reference)	6390 (92.8)
	Yes	494 (7.2)
PIVB infection	No (reference)	6697 (97.3)
	Yes	187 (2.7)

*First observed episodes of respiratory illness were analyzed for patients with multiple episodes during the nine-year study period. ‡Illness severity was informed by the type of healthcare service attended by the patient upon the collection of the first clinical specimen during a given episode of respiratory illness: General practitioner's surgery (GP), hospital (inpatients and outpatients), or intensive care unit (ICU; including also intensive therapy, high dependency, and coronary care units). ‡ Time with respect to the major circulation of the influenza A(H1N1)pdm09 virus in the UK: pre-pandemic, January 2005 – April 2009; pandemic, May 2009 – January 2011, Post-pandemic: February 2011 – December 2013.

Tables S6-S14: Binary logistic regression analysis. Negative and positive virus-virus interactions are highlighted in blue and red respectively. CI=confidence interval. [*]GP: general practice; Hospital: inpatients and outpatients; Intensive care: patients admitted to intensive care, intensive therapy, high-dependency, or coronary care units. [‡]Pre-pandemic: January 2005 to April 2009; pandemic (major circulation influenza A(H1N1)pdm09 virus in the UK): May 2009 to February 2011; post-pandemic: March 2011 to December 2013; [§]Infection and testing frequencies adjusting for response virus seasonality; IAV=influenza A virus [H1N1 and H3N2]; IBV=influenza B virus; RV=human rhinoviruses; CoV=human coronaviruses; MPV=human metapneumovirus; RSV=human respiratory syncytial virus [orthopneumovirus]; AdV=human adenoviruses [mastadenoviruses A-G]; PIVA= human respiroviruses [PIV1 and PIV3]; PIVB= human rubulaviruses [PIV2 and PIV4].

Table S6: Results of logistic regression modelling investigating virus interactions with IAV. Based on N=6,153 patients. Area under ROC curve = 78.64% (95% CI: 73.24% – 84.05%).

Explanatory variable	Levels	Odds ratio	95% CI	p-value
Age group (years)	1-5 (reference)	-	-	-
	<1	0.52	0.23-1.09	0.094
	6-16	0.41	0.12-1.11	0.113
	17-45	0.95	0.46-1.89	0.878
	46-64	0.8	0.36-1.68	0.566
	≥65	0.94	0.4-2.07	0.889
Sex	Female	-	-	-
	Male	1.66	1.03-2.74	0.042
Illness severity[*]	GP (reference)	-	-	-
	Hospital	0.8	0.46-1.43	0.435
	Intensive care	0.82	0.26-2.1	0.697
Pandemic period[‡]	Pre-pandemic (reference)	-	-	-
	Pandemic	0.73	0.3-1.62	0.456
	Post-pandemic	0.69	0.34-1.39	0.296
IAV infection freq[§]	-	3.33	2.37-4.76	<0.001
IAV testing freq[§]	-	0.8	0.41-1.63	0.528
IBV infection	No (reference)	-	-	-
	Yes	1.02	0.36-2.57	0.969
RV infection	No (reference)	-	-	-
	Yes	0.43	0.17-0.94	0.048
CoV infection	No (reference)	-	-	-
	Yes	0.97	0.4-2.09	0.938
MPV infection	No (reference)	-	-	-
	Yes	0.65	0.21-1.65	0.408
RSV infection	No (reference)	-	-	-
	Yes	1.21	0.5-2.63	0.649
AdV infection	No (reference)	-	-	-
	Yes	2.08	0.89-4.39	0.069
PIVA infection	No (reference)	-	-	-
	Yes	0.6	0.13-1.83	0.427
PIVB infection	No (reference)	-	-	-
	Yes	0.91	0.14-3.35	0.903

Table S7: Results of logistic regression modelling investigating virus interactions with IBV. Based on N=6,362 patients. Area under ROC curve = 81.98% (95% CI: 75.51% – 88.46%).

Explanatory variable	Levels	Odds ratio	95% CI	p-value
Age group (years)	1-5 (reference)	-	-	-
	<1	0.37	0.09-1.17	0.126
	6-16	1.75	0.72-4.1	0.202
	17-45	0.37	0.13-0.95	0.045
	46-64	0.67	0.27-1.62	0.385
	≥65	0.52	0.16-1.45	0.237
Sex	Female	-	-	-
	Male	0.79	0.43-1.41	0.418
Illness severity*	GP (reference)	-	-	-
	Hospital	0.37	0.19-0.7	0.002
	Intensive care	0.8	0.26-2.09	0.675
Pandemic period‡	Pre-pandemic (reference)	-	-	-
	Pandemic	0.96	0.31-2.96	0.945
	Post-pandemic	1.1	0.4-3.22	0.858
IBV infection freq§	-	3.09	1.87-5.31	<0.001
IBV testing freq§	-	1.11	0.45-2.92	0.824
IAV infection	No (reference)	-	-	-
	Yes	0.84	0.21-2.65	0.784
RV infection	No (reference)	-	-	-
	Yes	0.65	0.18-1.79	0.45
CoV infection	No (reference)	-	-	-
	Yes	1.31	0.35-3.81	0.649
MPV infection	No (reference)	-	-	-
	Yes	0.95	0.2-3.16	0.937
RSV infection	No (reference)	-	-	-
	Yes	0.23	0.03-0.94	0.078
AdV infection	No (reference)	-	-	-
	Yes	1.68	0.46-4.69	0.368
PIVA infection	No (reference)	-	-	-
	Yes	1.08	0.23-3.78	0.912
PIVB infection	No (reference)	-	-	-
	Yes	2.01	0.35-7.68	0.364

Table S8: Results of logistic regression modelling investigating virus interactions with RV. Based on N=4,629 patients. Area under ROC curve = 78.81% (95% CI: 76.66% – 80.96%).

Explanatory variable	Levels	Odds ratio	95% CI	p-value
Age group (years)	1-5 (reference)	-	-	-
	<1	1.78	1.39-2.3	<0.001
	6-16	0.45	0.27-0.71	0.001
	17-45	0.35	0.23-0.53	<0.001
	46-64	0.31	0.19-0.48	<0.001
	≥65	0.3	0.18-0.48	<0.001
Sex	Female	-	-	-
	Male	1.04	0.84-1.28	0.747
Illness severity*	GP (reference)	-	-	-
	Hospital	2.19	1.58-3.09	<0.001
	Intensive care	1.25	0.73-2.09	0.411
Pandemic period‡	Pre-pandemic (reference)	-	-	-
	Pandemic	1.51	1.08-2.11	0.016
	Post-pandemic	1.7	1.29-2.25	<0.001
RV infection freq§	-	1.49	1.19-1.88	0.001
RV testing freq§	-	0.71	0.58-0.86	<0.001
IAV infection	No (reference)	-	-	-
	Yes	0.27	0.14-0.51	<0.001
IBV infection	No (reference)	-	-	-
	Yes	0.41	0.2-0.78	0.01
CoV infection	No (reference)	-	-	-
	Yes	1.16	0.77-1.71	0.478
MPV infection	No (reference)	-	-	-
	Yes	1.01	0.64-1.56	0.974
RSV infection	No (reference)	-	-	-
	Yes	1.16	0.78-1.7	0.462
AdV infection	No (reference)	-	-	-
	Yes	1.6	1.09-2.31	0.014
PIVA infection	No (reference)	-	-	-
	Yes	0.88	0.56-1.34	0.567
PIVB infection	No (reference)	-	-	-
	Yes	1.27	0.73-2.12	0.383

Table S9: Results of logistic regression modelling investigating virus interactions with CoV. Based on N=6,274 patients. Area under ROC curve = 75.16% (95% CI: 71.71 – 78.61%).

Explanatory variable	Levels	Odds ratio	95% CI	p-value
Age group (years)	1-5 (reference)	-	-	-
	<1	1.44	0.98-2.13	0.064
	6-16	0.58	0.28-1.1	0.113
	17-45	0.68	0.4-1.14	0.155
	46-64	0.84	0.49-1.39	0.5
	≥65	0.74	0.38-1.33	0.329
Sex	Female	-	-	-
	Male	1.37	1.01-1.86	0.047
Illness severity*	GP (reference)	-	-	-
	Hospital	1.01	0.69-1.51	0.947
	Intensive care	0.91	0.46-1.7	0.775
Pandemic period‡	Pre-pandemic (reference)	-	-	-
	Pandemic	0.99	0.67-1.45	0.944
	Post-pandemic	0.45	0.28-0.72	0.001
CoV infection freq§	-	2.13	1.62-2.81	<0.001
CoV testing freq§	-	1.19	0.77-1.85	0.448
IAV infection	No (reference)	-	-	-
	Yes	0.6	0.29-1.15	0.139
IBV infection	No (reference)	-	-	-
	Yes	0.72	0.32-1.51	0.413
RV infection	No (reference)	-	-	-
	Yes	1.21	0.77-1.88	0.396
MPV infection	No (reference)	-	-	-
	Yes	1.03	0.51-1.92	0.933
RSV infection	No (reference)	-	-	-
	Yes	1.69	1.03-2.68	0.032
AdV infection	No (reference)	-	-	-
	Yes	1.92	1.16-3.07	0.008
PIVA infection	No (reference)	-	-	-
	Yes	2.09	1.14-3.67	0.013
PIVB infection	No (reference)	-	-	-
	Yes	0.67	0.16-1.87	0.505

Table S10: Results of logistic regression modelling investigating virus interactions with MPV. Based on N=6,452 patient. Area under ROC curve = 72.49% (95% CI: 67.75% – 77.22%).

Explanatory variable	Levels	Odds ratio	95% CI	p-value
Age group (years)	1-5 (reference)	-	-	-
	<1	0.87	0.52-1.44	0.596
	6-16	0.46	0.18-0.99	0.066
	17-45	0.22	0.08-0.5	0.001
	46-64	0.46	0.21-0.93	0.038
	≥65	0.72	0.35-1.38	0.337
Sex	Female	-	-	-
	Male	0.95	0.64-1.42	0.807
Illness severity*	GP (reference)	-	-	-
	Hospital	1.45	0.81-2.8	0.241
	Intensive care	1.42	0.55-3.46	0.454
Pandemic period‡	Pre-pandemic (reference)	-	-	-
	Pandemic	0.65	0.33-1.24	0.21
	Post-pandemic	0.62	0.37-1.04	0.068
MPV infection freq§	-	2.07	1.45-2.96	<0.001
MPV testing freq§	-	1	0.66-1.55	0.987
IAV infection	No (reference)	-	-	-
	Yes	0.4	0.13-1.03	0.077
IBV infection	No (reference)	-	-	-
	Yes	0.5	0.13-1.45	0.242
RV infection	No (reference)	-	-	-
	Yes	1.12	0.59-2.04	0.709
CoV infection	No (reference)	-	-	-
	Yes	1.03	0.47-2.06	0.944
RSV infection	No (reference)	-	-	-
	Yes	0.62	0.27-1.26	0.214
AdV infection	No (reference)	-	-	-
	Yes	1.63	0.84-2.97	0.13
PIVA infection	No (reference)	-	-	-
	Yes	0.76	0.28-1.72	0.539
PIVB infection	No (reference)	-	-	-
	Yes	1.4	0.4-3.75	0.548

Table S11: Results of logistic regression modelling investigating virus interactions with RSV. Based on N=6,198 patients. Area under ROC curve = 81.15% (95% CI: 78.34% – 83.96%).

Explanatory variable	Levels	Odds ratio	95% CI	p-value
Age group (years)	1-5 (reference)	-	-	-
	<1	1.67	1.21-2.33	0.002
	6-16	0.24	0.11-0.48	<0.001
	17-45	0.21	0.11-0.36	<0.001
	46-64	0.3	0.17-0.51	<0.001
	≥65	0.3	0.16-0.52	<0.001
Sex	Male	-	-	-
	Female	0.91	0.69-1.2	0.499
Illness severity*	GP (reference)	-	-	-
	Hospital	1.43	0.96-2.19	0.089
	Intensive care	1.69	0.94-3.02	0.077
Pandemic period‡	Pre-pandemic (reference)	-	-	-
	Pandemic	0.95	0.64-1.42	0.813
	Post-pandemic	0.74	0.51-1.07	0.111
RSV infection freq§	-	2.65	2.13-3.33	<0.001
RSV testing freq§	-	1.03	0.71-1.49	0.868
IAV infection	No (reference)	-	-	-
	Yes	0.82	0.42-1.54	0.546
IBV infection	No (reference)	-	-	-
	Yes	0.19	0.03-0.64	0.025
RV infection	No (reference)	-	-	-
	Yes	1.65	1.06-2.54	0.025
CoV infection	No (reference)	-	-	-
	Yes	1.86	1.16-2.91	0.008
MPV infection	No (reference)	-	-	-
	Yes	0.73	0.36-1.38	0.358
AdV infection	No (reference)	-	-	-
	Yes	1.21	0.75-1.9	0.417
PIVA infection	No (reference)	-	-	-
	Yes	1.58	0.87-2.75	0.118
PIVB infection	No (reference)	-	-	-
	Yes	1.07	0.45-2.26	0.867

Table S12: Results of logistic regression modelling investigating virus interactions with AdV. Based on N=6,449 patients. Area under ROC curve = 79.58% (95% CI: 76.82% – 82.33%).

Explanatory variable	Levels	Odds ratio	95% CI	p-value
Age group (years)	1-5 (reference)	-	-	-
	<1	0.94	0.69-1.27	0.697
	6-16	0.21	0.1-0.39	<0.001
	17-45	0.12	0.06-0.21	<0.001
	46-64	0.16	0.09-0.29	<0.001
	≥65	0.05	0.01-0.14	<0.001
Sex	Female	-	-	-
	Male	1.05	0.8-1.39	0.717
Illness severity*	GP (reference)	-	-	-
	Hospital	1.67	1.09-2.65	0.024
	Intensive care	1.03	0.5-2.04	0.934
Pandemic period‡	Pre-pandemic (reference)	-	-	-
	Pandemic	0.8	0.51-1.23	0.317
	Post-pandemic	0.73	0.51-1.06	0.098
AdV infection freq§	-	1.64	1.28-2.11	<0.001
AdV testing freq§	-	0.86	0.64-1.14	0.292
IAV infection	No (reference)	-	-	-
	Yes	1.19	0.61-2.2	0.603
IBV infection	No (reference)	-	-	-
	Yes	0.8	0.31-1.77	0.602
RV infection	No (reference)	-	-	-
	Yes	1.69	1.11-2.53	0.012
CoV infection	No (reference)	-	-	-
	Yes	1.74	1.07-2.75	0.021
MPV infection	No (reference)	-	-	-
	Yes	1.44	0.82-2.44	0.189
RSV infection	No (reference)	-	-	-
	Yes	0.82	0.51-1.3	0.422
PIVA infection	No (reference)	-	-	-
	Yes	1.66	0.97-2.73	0.053
PIVB infection	No (reference)	-	-	-
	Yes	2.46	1.26-4.49	0.005

Table S13: Results of logistic regression modelling investigating virus interactions with PIVA. Based on N=6,495 patients. Area under ROC curve = 75.94% (95% CI: 71.58% – 80.30%).

Explanatory variable	Levels	Odds ratio	95% CI	p-value
Age group (years)	1-5 (reference)	-	-	-
	<1	1.06	0.67-1.68	0.794
	6-16	0.06	0-0.3	0.007
	17-45	0.31	0.14-0.64	0.003
	46-64	0.41	0.18-0.82	0.018
	≥65	0.35	0.13-0.79	0.02
Sex	Female	-	-	-
	Male	1.25	0.84-1.88	0.282
Illness severity*	GP (reference)	-	-	-
	Hospital	1.23	0.69-2.35	0.502
	Intensive care	0.82	0.28-2.1	0.686
Pandemic period‡	Pre-pandemic (reference)	-	-	-
	Pandemic	0.96	0.52-1.73	0.895
	Post-pandemic	1.09	0.65-1.85	0.744
AdV infection freq§	-	1.68	1.18-2.37	0.004
AdV testing freq§	-	0.59	0.41-0.86	0.005
IAV infection	No (reference)	-	-	-
	Yes	0.51	0.12-1.55	0.294
IBV infection	No (reference)	-	-	-
	Yes	1.19	0.33-3.38	0.76
RV infection	No (reference)	-	-	-
	Yes	1.3	0.74-2.21	0.341
CoV infection	No (reference)	-	-	-
	Yes	2.39	1.25-4.35	0.006
MPV infection	No (reference)	-	-	-
	Yes	1.04	0.4-2.3	0.934
RSV infection	No (reference)	-	-	-
	Yes	1.42	0.73-2.62	0.282
AdV infection	No (reference)	-	-	-
	Yes	2.15	1.19-3.73	0.008
PIVB infection	No (reference)	-	-	-
	Yes	0.34	0.02-1.59	0.287

Table S14: Results of logistic regression modelling investigating virus interactions with PIVB. Based on N=6,743 patients. Area under ROC curve = 80.59% (95% CI: 75.31% – 85.88%).

Explanatory variable	Levels	Odds ratio	95% CI	p-value
Age group (years)	1-5 (reference)	-	-	-
	<1	0.55	0.24-1.15	0.123
	6-16	0.54	0.15-1.48	0.276
	17-45	0.45	0.15-1.17	0.129
	46-64	0.32	0.07-0.95	0.068
	≥65	0.13	0.01-0.62	0.045
Sex	Female	-	-	-
	Male	0.87	0.48-1.59	0.654
Illness severity*	GP (reference)	-	-	-
	Hospital	5.8	1.7-36.4	0.018
	Intensive care	4.58	0.73-35.66	0.102
Pandemic period‡	Pre-pandemic (reference)	-	-	-
	Pandemic	0.94	0.35-2.34	0.898
	Post-pandemic	0.78	0.35-1.75	0.539
AdV infection freq§	-	2.43	1.39-4.22	0.002
AdV testing freq§	-	0.99	0.54-1.85	0.987
IAV infection	No (reference)	-	-	-
	Yes	0.89	0.13-3.62	0.891
IBV infection	No (reference)	-	-	-
	Yes	2.5	0.51-9.07	0.199
RV infection	No (reference)	-	-	-
	Yes	2.75	1.26-5.89	0.009
CoV infection	No (reference)	-	-	-
	Yes	1.01	0.23-3.07	0.99
MPV infection	No (reference)	-	-	-
	Yes	2.02	0.56-5.63	0.223
RSV infection	No (reference)	-	-	-
	Yes	1.58	0.6-3.76	0.323
AdV infection	No (reference)	-	-	-
	Yes	4	1.83-8.26	<0.001
PIVA infection	No (reference)	-	-	-
	Yes	0.45	0.02-2.16	0.433

Table. S15: Odds ratios estimates from tests of 72 pairwise interactions generated from nine virus logistic regression models

		Explanatory virus								
		IAV	IBV	RV	CoV	MPV	RSV	AdV	PIVA	PIVB
Response virus	IAV		1.02	0.43	0.97	0.65	1.21	2.08	0.6	0.91
	IBV	0.84		0.65	1.31	0.95	0.23	1.68	1.08	2.01
	RV	0.27	0.41		1.16	1.01	1.16	1.60	0.88	1.27
	CoV	0.60	0.72	1.21		1.03	1.69	1.92	2.09	0.67
	MPV	0.40	0.50	1.12	1.03		0.62	1.63	0.76	1.40
	RSV	0.82	0.19	1.65	1.86	0.73		1.21	1.58	1.07
	AdV	1.19	0.80	1.69	1.74	1.44	0.82		1.66	2.46
	PIVA	0.51	1.19	1.30	2.39	1.04	1.42	2.15		0.34
	PIVB	0.89	2.50	2.75	1.01	2.02	1.58	4.00	0.45	

Table S16: Uncorrected p-values of odds ratios estimated from tests of 72 pairwise interactions generated from nine logistic regression models

		Explanatory virus								
		IAV	IBV	RV	CoV	MPV	RSV	AdV	PIVA	PIVB
Response virus	IAV		0.969	0.048	0.938	0.408	0.649	0.069	0.427	0.903
	IBV	0.784		0.450	0.649	0.937	0.078	0.368	0.912	0.364
	RV	<0.001	0.010		0.478	0.974	0.462	0.014	0.567	0.383
	CoV	0.139	0.413	0.396		0.933	0.032	0.008	0.013	0.505
	MPV	0.077	0.242	0.709	0.944		0.214	0.130	0.539	0.548
	RSV	0.546	0.025	0.025	0.008	0.358		0.417	0.118	0.867
	AdV	0.603	0.602	0.012	0.021	0.189	0.422		0.053	0.005
	PIVA	0.294	0.760	0.341	0.006	0.934	0.282	0.008		0.287
	PIVB	0.891	0.199	0.009	0.990	0.223	0.323	<0.001	0.433	

Table S17: P-values corrected using Holm's method to account for multiple hypothesis testing

		Explanatory virus								
		IAV	IBV	RV	CoV	MPV	RSV	AdV	PIVA	PIVB
Response virus	IAV		1	1	1	1	1	1	1	1
	IBV	1		1	1	1	1	1	1	1
	RV	<0.001	0.640		1	1	1	0.854	1	1
	CoV	1	1	1		1	1	0.544	0.806	1
	MPV	1	1	1	1		1	1	1	1
	RSV	1	1	1	0.544	1		1	1	1
	AdV	1	1	0.756	1	1	1		1	0.350
	PIVA	1	1	1	0.414	1	1	0.544		1
	PIVB	1	1	0.585	1	1	1	<0.001	1	

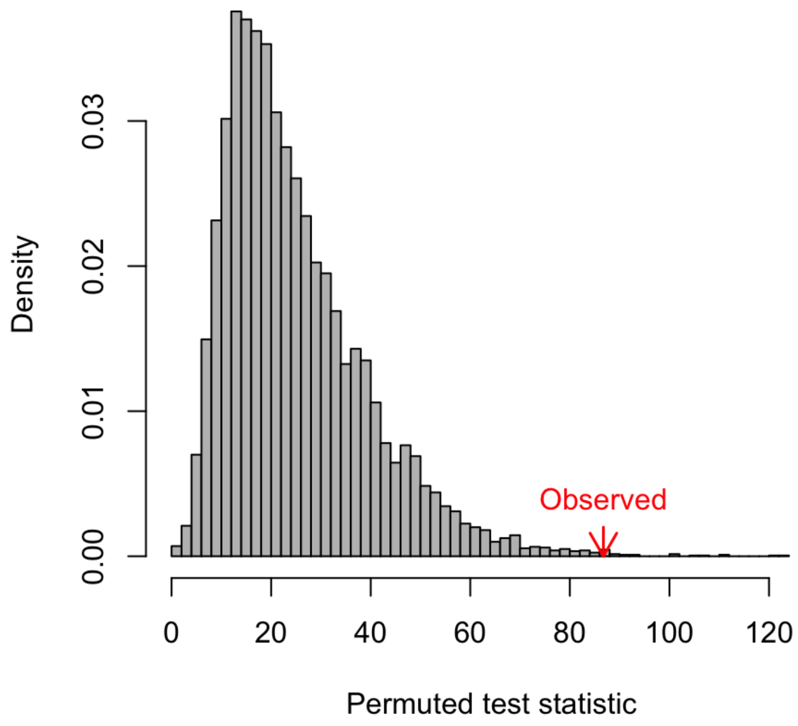

Fig. S2: Histogram illustrating a global test of pairwise interactions among the five virus groups (IBV, CoV, MPV, RSV, PIVA) where no significant individual pairwise interaction was found. The distribution of the test statistic, calculated using Fisher's method of combining p-values ($-2 \Sigma \log(p)$), was simulated under the global null hypothesis using 10,000 permutations (grey bars) and compared with the observed test statistic (red arrow), revealing strong evidence ($p=0.0021$) of association among the five virus groups.

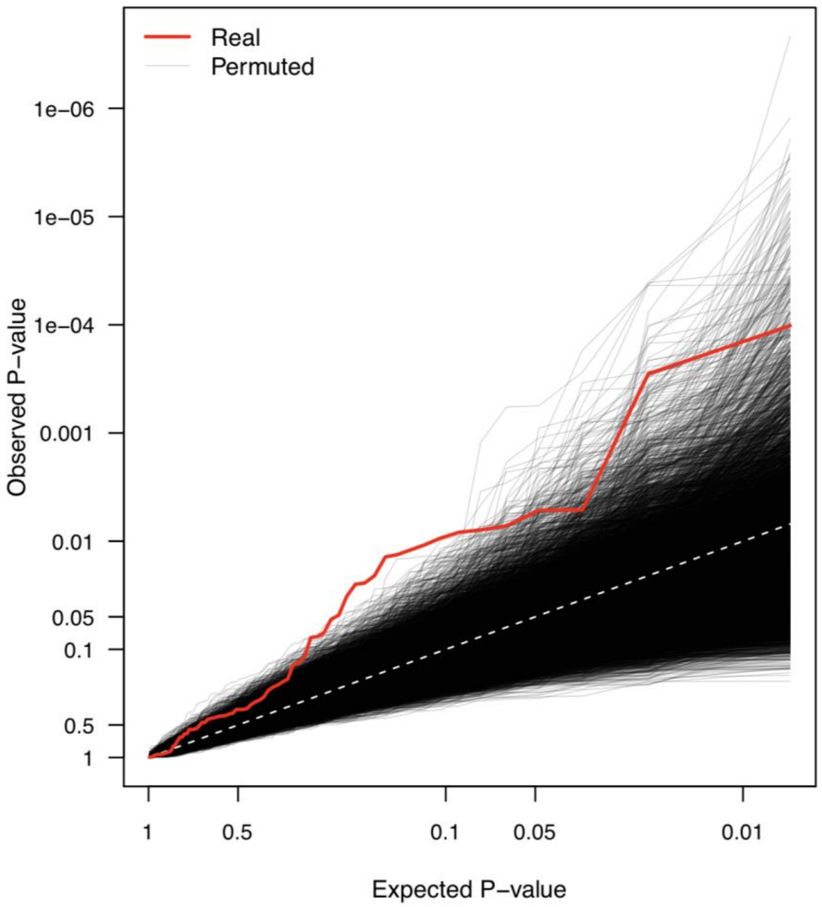

Fig. S3: Quantile-quantile (QQ) plot of the observed p-value distribution from the 72 pairwise tests among nine groups of respiratory virus (red line), compared with the expected QQ line under the global null hypothesis (white dashed line), and the distribution of QQ lines simulated from the global null hypothesis using 10,000 permutations (thin black lines). The steeper slope of the observed QQ line relative to the great majority of the permuted lines indicates that the observed p-values are lower than expected, providing strong evidence against the global null hypothesis of no association among any of the viruses ($p = 0.0003$; permutation test comparing the observed p-values from the observed data, combined using Fisher's method, to the 10,000 permuted data sets).

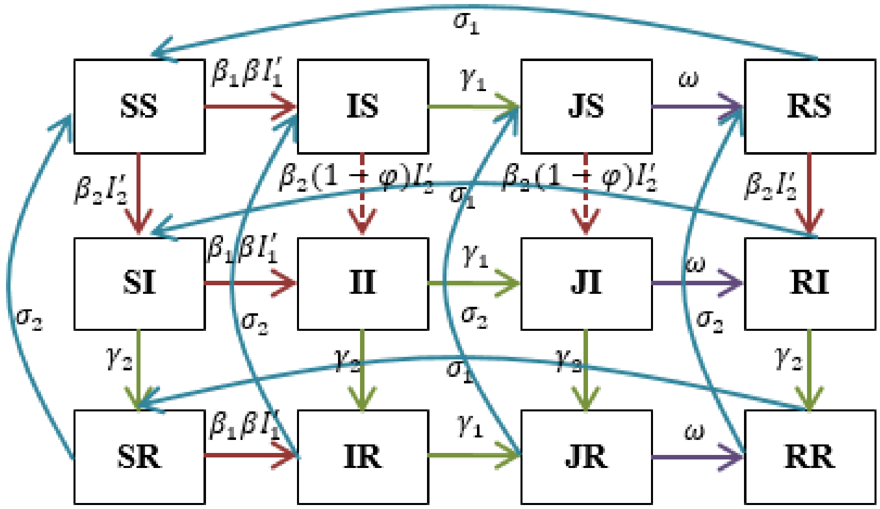

Fig. S4: Two-pathogen deterministic infection transmission model
Model class {1,2} denotes the infection status of individuals with respect to a seasonal influenza (virus 1) and a non-seasonal ubiquitous common cold virus (virus 2); subscripts 1 and 2 denote virus specific parameters; S=susceptible; I=infectious refractory phase; J=non-infectious refractory phase, R=immunity phase. See Table S17 for full details of model parameters.

Table S18: Two-pathogen mechanistic model parameter values and ranges

Parameter	Description	Value/ranges
B	Rate of virus 1 transmission/person	1 (days^{-1})
B	Seasonal amplitude of virus 1 forcing parameter	0.1
φ	Interaction parameter determining level of susceptibility to virus 2	0 - 1
β_1	Rate of virus 1 transmission/person	0.8 (days^{-1})
β_2	Rate of virus 2 transmission/person	1 (days^{-1})
γ_1	Rate of transition to non-infectious (i.e. 1/infectious period)	0.5 (days^{-1})
γ_2	Rate of transition to non-infections (i.e. 1/infectious period)	0.5 (days^{-1})
ω	Rate at which individuals acquire antibodies	0.04 - 1 (days^{-1})
σ_1	Rate of waning immunity against virus 1	0.0027 (days^{-1})
σ_2	Rate of waning immunity against virus 2	0.0274 (days^{-1})
μ	Non-disease-induced mortality rate (1/life expectancy)	1/(70*365) (days^{-1})

Table S19: Investigating the sensitivity of patient coinfection prevalence to the time frame window used to define an episode of respiratory illness.

Episode window (days)	(A) Number of patient episodes of illness	(B) Number of patient episodes tested for all viruses	(C) Number of virus-positive patient episodes tested for all viruses	% coinfected (among B)	% coinfected (among C)
1	49,844	31,353	10,737	3.5	10.2
10	46,350	28,851	10,049	3.7	10.6
20	45,076	27,916	9,812	3.8	10.8
**30**	**44,230**	**27,284**	**9,654**	**4.0**	**11.2**
40	43,690	26,865	9,570	4.1	11.5
50	43,212	26,508	9,494	4.3	12.0
60	42,852	26,244	9,428	4.5	12.5

*** The episode window used in the analyses is highlighted in bold.**

References

1. Mair C, *et al.* (2019) Estimation of temporal covariances in pathogen dynamics using Bayesian multivariate autoregressive models. In Press. *Plos Computational Biology*.
